# Comparative efficacy and safety of low-power versus high-power holmium laser enucleation of the prostate for benign prostatic hyperplasia: an updated meta-analysis

**DOI:** 10.1097/MS9.0000000000004950

**Published:** 2026-04-14

**Authors:** Muhammad Saeed Qazi, Sanya Ashraf, Najaf Ahmed Rajpar, Shree Rath, Sabahat Ul Ain Munir Abbasi, Arisha Akhtar, Neha Malik, Muskan Kumari, Ahmed Mueed, Fnu Farukhuddin, Muhammad Riyyan, Amina Rizwan, Bakhtawar Rani, Ghulam Muhammad, Muhammad Muzamil Rafique, Muhammad Usman, Pratik Bhattarai

**Affiliations:** a Department of Medicine, Bilawal Medical College, Liaquat University of Medical and Health Sciences, Jamshoro, Pakistan; b Department of Medicine, Liaquat University of Medical and Health Sciences, Jamshoro, Pakistan; c Department of Medicine, All India Institute of Medical Sciences, Bhubaneswar, India; d Department of Medicine, Allama Iqbal Medical College, Lahore, Pakistan; e Department of Dentistry, Ziauddin University, Karachi, Pakistan; f Department of Medicine, Fatima Memorial College of Medicine and Dentistry, Lahore, Pakistan; g Department of Internal Medicine, Warren Alpert Medical School, Brown University, Rhode Island, United States; h Department of Science, Bay Path University, Massachusetts, United States; i Department of Medicine, Khairpur Medical College, Khairpur Mir, Pakistan; j Department of Medicine, Federal Medical College, Islamabad, Pakistan; k Department of Medicine, Manipal College of Medical Sciences, Pokhara, Nepal

**Keywords:** benign prostatic hyperplasia, high-power HoLEP, low-power HoLEP, urinary outcomes

## Abstract

**Background:**

Holmium laser enucleation of the prostate (HoLEP) is the gold-standard surgical treatment for benign prostatic hyperplasia, irrespective of the prostate size. However, the optimal laser power setting remains uncertain. High-power systems (80–100 W) are commonly used in clinical practice, while emerging evidence suggests that low-power settings (24–50 W) may offer comparable efficacy with potential functional and safety advantages.

**Objective:**

To compare the perioperative, functional, and safety outcomes of low-power versus high-power HoLEP based on randomized controlled trials.

**Methods:**

We conducted a systematic review and meta-analysis of RCT comparing low-power (24–50 W) and high-power (80–100 W) HoLEP, searching four electronic databases from inception till April 2025. The data extracted on primary outcomes include International Prostate Symptom Score (IPSS), maximum urinary flow rate (Qmax), post-void residual volume (PVR), quality of life (QoL), and Clavien–Dindo-graded complications. Secondary outcomes included operative efficiency metrics. A random-effects model was used to calculate pooled effect sizes with 95% confidence intervals. Risk of bias was assessed using the Cochrane risk of bios (RoB 2) tool.

**Results:**

Four trials (*n* = 394) were included. Low-power and high-power HoLEP showed similar IPSS improvement [MD: −0.30 (95% CI: −1.00 to 0.41)] and Qmax (0.30 ml/s, −0.42 to 1.01). Low-power HoLEP resulted in better QoL [MD: 0.20 (95% CI: 0.10–0.31] and reduced PVR (–2.33 ml, −4.09 to −0.58). High-power HoLEP had shorter operative (–12.62 min) and enucleation times (–6.89 min). Safety outcomes revealed no significant differences.

**Conclusion:**

Low-power HoLEP is clinically non-inferior, providing comparable symptom relief and urinary flow improvement with better quality-of-life outcomes and more complete bladder emptying. Although operative times are modestly longer, the safety profile remains equivalent. These findings support low-power HoLEP as a viable surgical option.

## Introduction

Benign prostatic hyperplasia (BPH) is a complex progressive condition of aging men with significant implications for quality of life (QoL) and health care systems. It is found to affect around 50–60% of men by the time they reach 60 years of age and around 80–90% of men by the time they reach 70 years of age^[^1^]^. BPH is characterized by lower urinary tract symptoms (LUTS) including urinary urgency, increased frequency, weak stream, and nocturia. It contributes to bladder outlet obstruction and, if left untreated, can cause urinary retention, recurrent infections, and even renal dysfunction^[^2^]^. Holmium laser enucleation of the prostate (HoLEP) has emerged as a new highly effective, size-independent, minimally invasive endoscopic gold standard for surgical BPH treatment offering durable symptomatic relief, better hemostasis, shorter hospitalization time, and reduced complication rates compared to traditional transurethral resection of the prostate (TURP)^[^3^]^.

Over the past decade, technological advancements have enabled variations in laser power settings, giving rise to a clinical debate regarding the comparative benefits of low-power HoLEP (LP-HoLEP) versus high-power HoLEP (HP-HoLEP) techniques. Traditionally, high-power settings have been favored for their efficiency in tissue ablation and hemostasis; however, emerging evidence suggests that low-power settings can be as effective with less thermal damage, less postoperative dysuria, and better efficiency^[^4,5^]^. Given the increasing adoption of LP-HoLEP in clinical practice, a rigorous, updated analysis is warranted to determine whether reduced laser power compromises treatment outcomes or enhances patient safety.

This meta-analysis aims to comprehensively compare the efficacy and safety of LP- vs HP-HoLEP in BPH patients. We systematically reviewed randomized control trials presenting important clinical outcomes such as the International Prostate Symptom Score (IPSS), maximum urinary flow rate (Qmax), post-void residual volume (PVR), and multiple perioperative and postoperative parameters. Using this evidence synthesis, we seek to bring clarity to urologists when dealing with laser settings in HoLEP and inform clinical decision-making with evidence-based insight into which modality of power best meets efficacy, safety, and patient satisfaction.

In line with the TITAN 2025 guidelines^[^6^]^ on declaring AI usage in research and manuscript preparation, we affirm that no generative AI tools were employed in the conceptualization, drafting, or revision of this manuscript.

## Methods

### Protocol

This updated systematic review and meta-analysis was conducted in accordance with the *Cochrane Handbook for Systematic Reviews of Interventions*^[^7^]^ and the *Preferred Reporting Items for Systematic Reviews and Meta-Analyses (PRISMA)*^[^8^]^ guidelines to ensure transparency and international reliance.

### Data sources and search strategy

Two independent reviewers conducted a comprehensive literature search across four electronic databases: PubMed, Scopus, Google Scholar, and the Cochrane Central from their inception until 5 April 2025, to identify relevant original full-text articles. No restrictions for language were used. The search strategy was based on the MeSH terms and free-text keywords, organized according to the PICO (Population, Intervention, Comparison, and Outcome) framework. Detailed search strategies for each database are included in Supplemental Digital Content Table 1, available at: http://links.lww.com/MS9/B117. All records retrieved from Google Scholar were manually screened, and only peer-reviewed journal articles were considered eligible. Grey literature, including conference abstracts, theses, dissertations, reports, preprints, and other non–peer reviewed material, was excluded. Additionally, a manual search of the reference lists of the included studies was performed to identify any further relevant articles.

HIGHLIGHTS

- Both low-power and high-power holmium laser enucleation of the prostate (HoLEP) achieved comparable improvements in International Prostate Symptom Score and urinary flow rate.
- Low-power HoLEP resulted in significantly better quality-of-life scores and reduced post-void residual urine volume.
- High-power HoLEP demonstrated shorter operative and enucleation times, indicating higher procedural efficiency.
- Low-power HoLEP is a clinically non-inferior, safe, and effective alternative to high-power systems, especially for preserving functional outcomes.

### Eligibility criteria

#### Inclusion criteria

Studies were considered eligible for inclusion if they met the following criteria: (1) they were randomized controlled trials (RCTs), (2) they involved human male patients diagnosed with BPH, (3) they included a minimum of 80 participants, (4) the intervention compared LP-HoLEP (24–50 W) with HP-HoLEP (80–100 W), and (5) they reported at least one of the following outcomes: IPSS, perioperative and postoperative parameters (such as operative time, hemoglobin drop, catheterization time, and hospital stay), Qmax, and PVR.

#### Exclusion criteria

Studies were excluded if: (1) they were animal studies or *in vitro* investigations, (2) they were reviews, systematic reviews, meta-analyses, case reports, case series, letters to the editor, editorials, brief communications, or conference abstracts, (3) they were non-randomized trials, observational studies, or quasi-experimental designs, and (4) they compared either intervention to placebo or used alternative surgical interventions other than low- and HP-HoLEP.

### Study selection

All references identified through the systematic search were exported to Rayyan.ai, where duplicates were screened and removed. Two reviewers (A.A. and N.M.) independently screened the titles and abstracts of the retrieved articles to assess their relevance based on the predefined inclusion and exclusion criteria. Full-text articles of potentially eligible studies were then reviewed in detail. Any disagreements between the reviewers were resolved through discussion or consultation with a third reviewer (N.A.R.).

### Data extraction

Data from the included studies were extracted by two independent reviewers (A.A. and M.S.Q.), and a third reviewer (N.M.) cross-checked the extracted information for accuracy and consistency. The extracted data included the *baseline characteristics*: study design, year of publication, sample size, country, patient age, baseline prostate-specific antigen (PSA) level, preoperative prostate volume (ml), duration of disease (months), comorbidities, energy settings, and type of enucleation performed; and the *outcome characteristics (extracted separately for both low-power and high-power groups*): study title and author, total IPSS score, Qmax (ml/s), stress urinary incontinence (SUI), voided urine volume (ml), post-void residual urine volume (PVR, ml), QoL score, storage symptom score, postoperative Visual Analogue Scale (VAS) for pain, PSA level (ng/ml) at baseline, total Overactive Bladder Symptom Score, incidence of urethral stricture, incidence of bladder neck contracture (BNC), irrigation fluid volume (l), morcellation time (minutes), enucleation time (minutes), hemoglobin decrease (g/dl), irrigation time (hours), total operation time (minutes), extracted tissue volume (ml), and catheterization time (hours).

### Risk of bias and quality assessment

The risk of bias was assessed by two independent authors (N.A.R. and M.S.Q.) using the Cochrane risk of bias tool (RoB 2)^[^10^]^. Discrepancies were resolved by a third reviewer (M.O.L.). The RoB 2 tool assesses the randomized control trials over five bias domains, including (1) bias due to the randomization process, (2) deviation from intended intervention, (3) missing outcome data, (4) measurement of outcomes, and (5) selection of the reported result, and “overall risk of bias” judgment. The studies were rated low risk, some concerns, and of high-risk quality based on judgment.

### Data synthesis and statistical analysis

A meta-analysis was performed using Review Manager (RevMan) version 5.4.0^[^11^]^ in accordance with PRISMA guidelines and Cochrane Collaboration recommendations^[^7,8^]^. For continuous outcomes, mean differences (MDs) with 95% confidence intervals (CIs) were calculated. A random-effects model using the DerSimonian and Laird method was applied to account for between-study variability. For dichotomous outcomes, pooled risk ratios (RRs) with corresponding 95% CIs were calculated using the Mantel–Haenszel method. Statistical heterogeneity was assessed using the *I*^2^ statistic, with thresholds of 25, 50, and 75% representing low, moderate, and high heterogeneity, respectively. For outcomes with *I*^2^ > 50%, sensitivity analyses were performed using the leave-one-out method: excluding one study at a time to explore the robustness of the findings^[^12^]^. Forest plots were generated for each outcome to visually represent the pooled estimates and 95% CIs. Due to the limited number of studies per outcome (<10), funnel plots or Egger’s regression test was not performed. A *P*-value of <0.05 was considered statistically significant.

## Results

### Literature search

**Figure 1.**
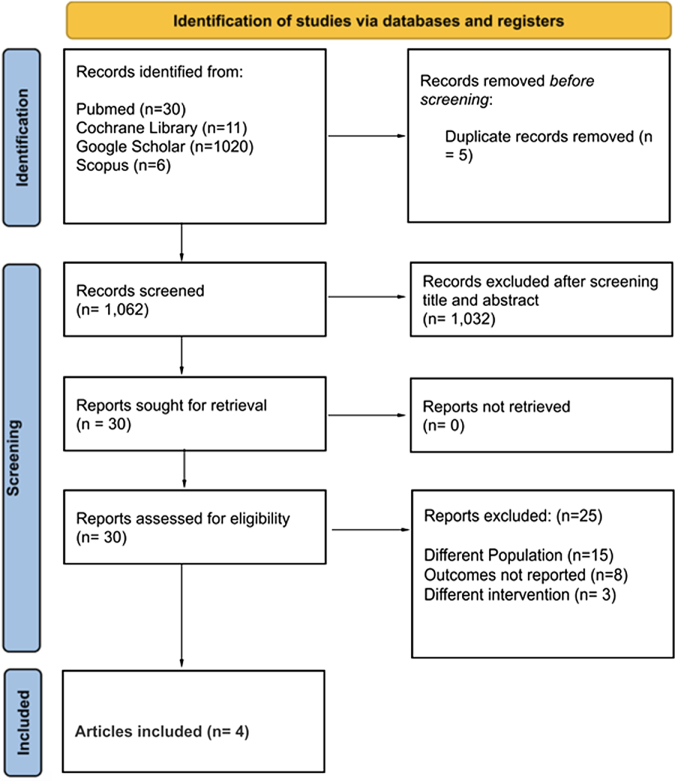
PRISMA flowchart of included studies.

Initial search revealed a total of 1067 potential articles. After removing duplicates, 1062 articles were screened. Following the exclusion of 1032 articles after title and abstract screening, 30 articles were assessed for eligibility. Twenty-five articles were excluded as they did not comply with the inclusion criteria. Finally, four articles were included in this meta-analysis consisting of all four randomized control trials. The PRISMA flowchart summarizing the screening process is given in Figure 1.

### Study characteristics

Our study included four RCTs comparing LP-HoLEP vs HP-HoLEP, with a total of 394 patients (196 in the low-power and 198 in the high-power groups). The average age of participants was 68.86 years in the low-power group and 68.26 years in the high-power group. Baseline characteristics were largely similar between groups, except for energy settings, which ranged from 24 W (2 J, 12 Hz) to 50 W (2 J, 50 Hz) in the low-power group and 80 W (2 J, 40 Hz) to 100 W (2.5 J, 40 Hz) in the high-power group, and preoperative prostate volumes, which ranged from 30.5 to 147 ml in the low-power group and 32.1 to 137.6 ml in the high-power group. There was considerable variation in the surgical techniques employed as well with some studies using *en bloc* enucleation/biplanar technique as outlined by Wang *et al*^[^13^]^, while others used two or three lobe enucleation. Baseline PSA levels were comparable, averaging 3.90 ng/ml in the low-power group and 3.94 ng/ml in the high-power group. Baseline characteristics are summarized in Table 1.

**Table 1:** Characteristics of included studies comparing low-power vs high-power holmium laser enucleation of the prostate.

Author, year of publication	Type of study	Mean age (±SD), low-power vs high-power	Enrolled patients, low-power vs high-power (total)	Surgical technique	Energy setting, low-power W (J, Hz)	Energy setting, high-power W (J, Hz)	Mean preoperative PV (±SD), ml, low-power vs high-power	Baseline PSA level (ng/ml), low-power vs high-power
Du *et al* (2025)	RCT	71.3 ± 1.7 vs 70.8 ± 1.9	49 vs 53 (109)	*En bloc*	37.5 W (1.5 J, 25 Hz)	100 W (2.5 J, 40 Hz)	99.7 ± 11.2 vs 100.1 ± 11.9	6.7 ± 1.4 vs 6.8 ± 1.5
Gao *et al* (2024)	RCT	66.6 ± 6.9 vs 65.7 5.4	40 vs 41 (81)	*En bloc*	24 W (1.2 J, 20 Hz)	80 W (2 J, 40 Hz)	30.5 ± 4.2 vs 32.1 ± 4.3	3.1 ± 1.4 vs 3.5 ± 1.2
Suh *et al* (2023)	RCT	71.5 ± 7.8 vs 69.3 ± 7.3	46 vs 44 (90)	Three lobe	24 W (2 J, 12 Hz)	80 W (2 J, 40 Hz)	64.3 ± 25.7 vs 67.1 ± 23.7	1.6 ± 3.1 vs 0.9 ± 0.8
Elshal *et al* (2018)	RCT	66.4 ± 7 vs 67 ± 7	61 vs 60 (121)	Two lobe or Three lobe^a^	50 W (2 J, 25 Hz)	100 W (2 J, 50 Hz)	147 ± 59 vs 137.6 ± 58	NR

Hz, Hertz; J, Joule; NR, not reported; PV, prostate volume; RCT, randomized controlled trial; W, Watt.

^a^ Enucleation was performed according to prostate morphology using either approach.

### Quality assessment

Risk of bias was assessed using ROB 2, which indicated that most studies had either a low risk of bias or some concerns. There were some concerns with Du *et al*^[^5^]^ and Gao *et al*^[^4^]^ regarding selection of reported results (D5). Only Suh *et al*^[14]^ was rated as having a high risk of bias, primarily due to concerns in the randomization process (D1), outcome measurement (D4), and some concern over selection of reported results. Overall, the included studies demonstrate a generally low to moderate risk of bias, supporting the reliability of the pooled findings (Supplemental Digital Content Figure 1, available at: http://links.lww.com/MS9/B117, Supplemental Digital Content Figure 2, available at: http://links.lww.com/MS9/B117).

### Study outcomes

#### Symptom improvement & patient-reported outcomes

##### Total IPSS

**Figure 2.**
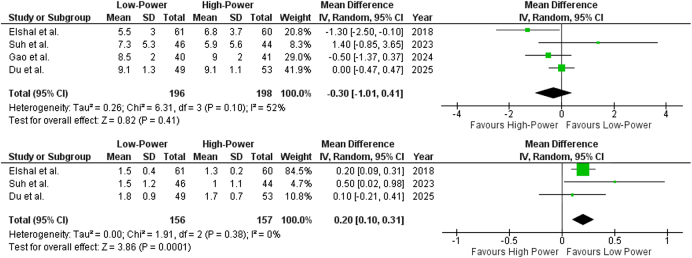
Forest plot comparing (**A**) Total International Prostate Symptom Score (IPSS) (*I*^2^ = 52%, *P* = 0.41) and (**B**) quality of life (QoL) score [*I*^2^ = 0%, *P* = 0.0001] between low-power and high-power HoLEP.

Data from all four included studies reported total IPSS. The pooled mean difference was −0.30 (95% CI: −1.00 to 0.41; *P* = 0.41) (Fig. 2A), indicating no statistically significant difference between the two groups. Heterogeneity analysis revealed moderate variability among studies (*I*^2^ = 52%), suggesting some inconsistency in effect sizes. Upon sensitivity analysis, removal of Elshal *et al*^[^15^]^ [MD: −0.07 (95% CI: 0.63–0.49; *P* = 0.81)] reduced heterogeneity from moderate to low (*I*^2^ = 25%; Supplemental Digital Content Figure 3, available at: http://links.lww.com/MS9/B117). The lack of prostate volume restrictions in Elshal *et al*^[^15^]^ is likely the primary contributor to heterogeneity, as prostate size directly influences baseline symptom severity (IPSS) and treatment response, leading to variability in pooled outcomes.

##### QoL score

Data from three out of four included studies reported QoL score. The pooled mean difference was 0.20 (95% CI: 0.10–0.31; *P* = 0.0001) (Fig. 2B), indicating a statistically significant difference favoring LP-HoLEP. Heterogeneity analysis showed no variability among studies (*I*^2^ = 0%), suggesting consistent effect sizes across the included studies.

#### Urinary function and flow dynamics

##### Qmax (ml/s)

**Figure 3.**
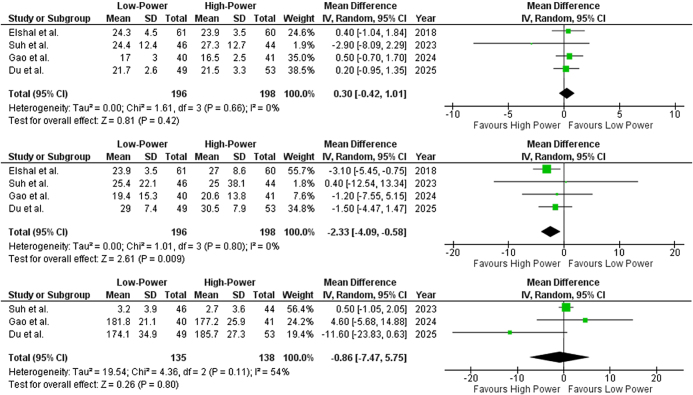
Forest plot of (**A**) maximum urinary flow rate (Qmax) (*I*^2^ = 0%, *P* = 0.42), (**B**) post-void urine volume (PVR) (*I*^2^ = 0, *P* = 0.009), and (**C**) voided urine volume (*I*^2^ = 54%, *P* = 0.80).

Data from all four included studies reported Qmax. The pooled mean difference was 0.30 (95% CI: −0.42 to 1.01; *P* = 0.42) (Fig. 3A), indicating no statistically significant difference among the two groups. Heterogeneity analysis showed no variability among studies (*I*^2^ = 0%), suggesting consistent effect sizes across the included studies.

##### PVR (ml)

Data from all four included studies reported post-void urine volume. The pooled mean difference was −2.33 (95% CI: −4.09 to −0.58; *P* = 0.009) (Fig. 3B), indicating a statistically significant reduction in PVR favoring LP-HoLEP. Heterogeneity was low (*I*^2^ = 0%), suggesting consistency across studies. These findings suggest that LP-HoLEP may provide more efficient bladder emptying than HP-HoLEP, potentially leading to better postoperative outcomes.

##### Voided urine volume (ml)

Data from three out of four included studies reported voided urine volume. The pooled mean difference was −0.86 (95% CI: −7.47 to 5.75; *P* = 0.80) (Fig. 3C), indicating no statistically significant difference among the two groups. Heterogeneity analysis revealed moderate variability among studies (*I*^2^ = 54%), suggesting some inconsistency in effect sizes. Upon sensitivity analysis, removal of Du *et al*^[^5^]^ [MD: 0.59 (95% CI: −0.94 to 2.12; *P* = 0.45) resolved the heterogeneity (*I*^2^ = 0%; Supplemental Digital Content Figure 4, available at: http://links.lww.com/MS9/B117) completely which may be explained by Du *et al*’s^[^5^]^ inclusion of only patients with a prostate volume >80 ml, as larger prostates tend to cause more severe bladder outlet obstruction, affecting baseline voided urine volume and its improvement after surgery.

#### Postoperative complications

##### SUI

**Figure 4.**
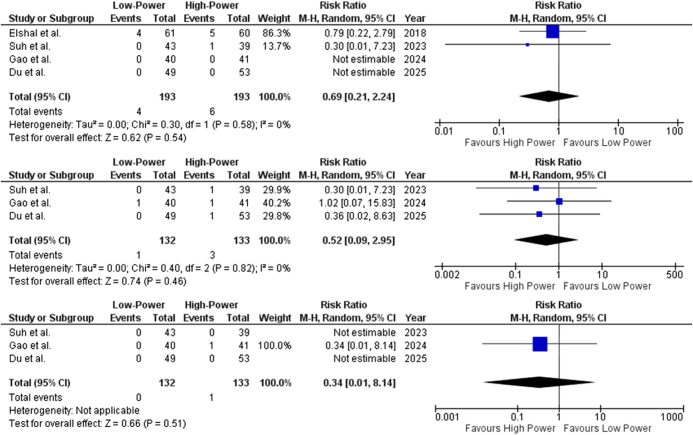
Forest plot of (**A**) stress urinary incontinence (SUI) (*I*^2^ = 0%, *P* = 0.54), (**B**) urethral strictures (*I*^2^ = 0%, *P* = 0.46), and (**C**) bladder neck contracture (*I*^2^ = N/A, *P* = 0.51).

Data from all four included studies reported the occurrence of SUI (Clavien–Dindo Grade I^[^7^]^). The pooled risk ratio for SUI following LP- vs HP-HoLEP was 0.69 (95% CI: 0.21–2.24; *P* = 0.54) (Fig. 4A), indicating no statistically significant difference between the two approaches. Low heterogeneity (*I*^2^ = 0%) suggests consistency across studies, but wide confidence intervals indicate uncertainty in the effect estimate.

##### Urethral strictures

Data from three out of four included studies reported the occurrence of urethral strictures (Clavien–Dindo Grade IIIa^[^7^]^). Pooled risk ratio of 0.52 (95% CI: 0.09–2.95; *P* = 0.46) (Fig. 4B) was obtained, suggesting no significant difference between the two techniques, though event rates were low, making estimates uncertain.

##### BNC

Data from three out of four included studies reported the occurrence of BNC (Clavien–Dindo Grade IIIb^[^16^]^). A pooled risk ratio of 0.34 (95% CI: 0.01–8.14; *P* = 0.51) (Fig. 4C) was obtained, suggesting no significant difference between the use of LP- and HP-HoLEP in the risk of developing BNC. However, results were largely non-estimable due to the absence of events in most studies, leading to considerable uncertainty in the effect estimate.

#### Perioperative and surgical efficiency metrics

##### Total operation time (min)

**Figure 5.**
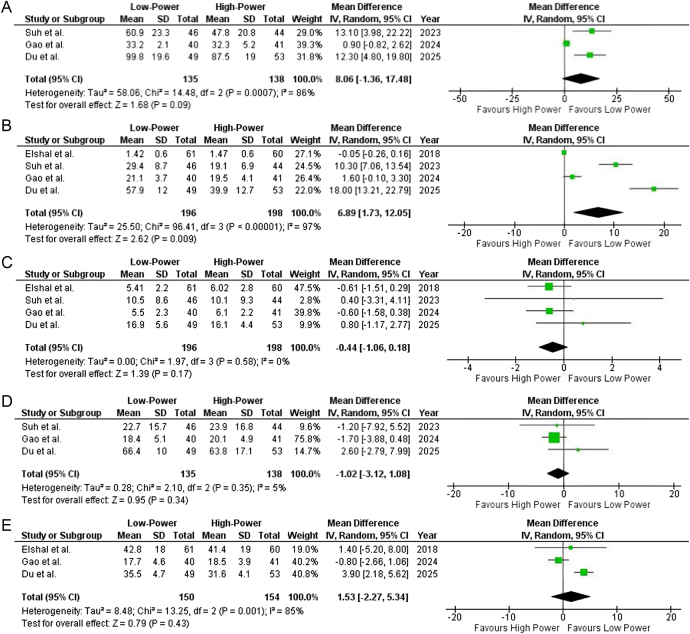
Forest plot of (**A**) total operation time (*I*^2^ = 86%, *P* = 0.09), (**B**) enucleation time (*I*^2^ = 97%, *P* = 0.009), (**C**) morcellation time (*I*^2^ = 0%, *P* = 0.17), (**D**) extracted tissue volume (*I*^2^ = 5%, *P* = 0.34), and (**E**) irrigation fluid volume (*I*^2^ = 85%, *P* = 0.43).

Data from three out of four included studies reported total operation time. The initial pooled mean difference was 8.06 (95% CI: −1.36 to 17.48; *P* = 0.09) (Fig. 5A) with substantial heterogeneity (*I*^2^ = 86%), likely due to Gao *et al*’s^[^4^]^ inclusion of patients with a prostate volume <40 ml, as smaller prostates require shorter enucleation and morcellation times, significantly impacting total operation time. Sensitivity analysis excluding Gao *et al*^[^4^]^ reduced heterogeneity to 0% (Supplemental Digital Content Figure 5, available at: http://links.lww.com/MS9/B117) and resulted in a statistically significant pooled mean difference of 12.62 (95% CI: 6.83–18.42; *P* < 0.0001), suggesting that low-power settings are associated with longer operation times.

##### Enucleation time (min)

Data from all four included studies reported enucleation time. The pooled mean difference was 6.89 (95% CI: 1.73–12.05; *P* = 0.009) (Fig. 5B), indicating a statistically significant difference among the two groups and suggesting that LP-HoLEP is associated with longer enucleation times compared with HP-HoLEP. There was considerable heterogeneity in the results (*I*^2^ = 97%), which could not be resolved using sensitivity analysis most likely due to differences in prostate volume inclusion criteria across studies. Larger prostates require longer enucleation times, and studies like Du *et al*^[^5^]^, which included only prostates >80 ml, likely reported longer enucleation times compared to studies like Gao *et al*^[^4^]^, which included <40 ml prostates. This variability in baseline prostate size significantly impacts procedural duration, contributing to unresolved heterogeneity (Supplemental Digital Content Figure 6, available at: http://links.lww.com/MS9/B117).

##### Morcellation time (min)

Data from all four included studies reported morcellation time. The pooled mean difference was −0.44 (95% CI: −1.06–0.18; *P* = 0.17) (Fig. 5C), suggesting no significant difference between low-power and high-power techniques. Heterogeneity was low (*I*^2^ = 0%), indicating consistent findings across studies.

##### Extracted tissue volume (ml)

Data from three out of four included studies reported extracted tissue volume. The pooled mean difference was −1.02 (95% CI: −3.12 to 1.08; *P* = 0.34) (Fig. 5D), suggesting no significant difference between low-power and high-power techniques. Heterogeneity was low (*I*^2^ = 5%), indicating consistent findings across studies.

##### Irrigation fluid volume (l)

Data from three studies reported irrigation fluid volume, with a pooled mean difference of 1.53 (95% CI: −2.27 to 5.34; *P* = 0.43) (Fig. 5E), indicating no significant difference between low-power and high-power techniques. However, heterogeneity was high (*I*^2^ = 85%), suggesting substantial variability across studies. Upon sensitivity analysis, excluding Gao *et al*^[^4^]^ and later Du *et al*^[^5^],^ due to their distinct patient population (smaller prostates <40 ml and larger prostates >80 ml), reduced heterogeneity to *I*^2^ = 0% (Supplemental Digital Content Figure 7, available at: http://links.lww.com/MS9/B117), suggesting that differences in baseline prostate size contributed significantly to the initial heterogeneity. The updated pooled analysis after removal of Gao *et al*^[^4^]^ [MD: 3.74 (95% CI: 2.08 to 5.40), *P* < 0.0001] indicates a statistically significant difference between two groups with LP-HoLEP associated with greater irrigation fluid volumes than HP-HoLEP.

## Discussion

This meta-analysis comprehensively assessed operative and patient-related outcomes following the use of either low-power or high-power LP-HoLEP and HP-HoLEP. Our analysis concluded no significant change in IPSS score between the groups. However, a significantly higher QoL score was noted among patients following the use of low-power laser enucleation. LP-HoLEP also resulted in reduced post-void urine volume, while other urinary function markers like maximum urine flow rate and voided urine volume were similar between both groups. Perioperative outcomes revealed a longer procedure time and enucleation time on use of low-power settings. The need for irrigation fluid was higher on low-power settings as well. Postoperative complications were similar across both groups.

The rationale of high- vs low-power settings results from an effort to balance efficacy with adverse events. High-power LEP is effective, particularly in patients with large prostate glands^[^17^]^. However, adverse events like retrograde ejaculation are more common^[^18^].^ On the other hand, low-power settings allow for effective hemostasis and dissection^[^19^]^.

Our analysis noted no difference in IPSS scores between the two groups. On the other hand, QoL scores were greatly improved following LP-HoLEP. This implies that, while no difference was noted in the overall symptoms experienced by patients in both groups, a subjective improvement was perceived by patients on the use of LP-HoLEP. This is in accordance with the findings by Schumacher *et al*, which reported 94% of patients experiencing better urination after HoLEP^[^20^]^. Sun *et al* noted that these improvements persist even on a 5-year follow-up, implying long-lasting improvements in LUTS symptoms^[^21^]^.

Analysis of urinary functions and flow dynamics revealed lower post-void urine volume in the low-power group, despite similar Qmax and voided urine volume in both groups. This implies that urinary retention was greatly reduced with the use of low-power settings. The lower energy settings may have resulted in lesser thermal injury of the adjacent structures, leading to less incidence of postoperative edema and inflammation^[^4^]^. The reduced irritative symptoms, pain, and dysuria may further result in more comfortable and complete voiding, resulting in lower post-void urine volumes^[^15^]^. Functional preservation of sphincters may be suggested through the lower PVR.

Evaluation of postoperative complications revealed no significant differences in stress urinary incontinence, urethral structures, and BNCs. This suggests there may be no difference in the immediate postoperative outcomes of patients. Outcomes were homogenous throughout, implying similar efficacy with certainty. Perioperative and surgical measures revealed longer operative and enucleation times on the use of low-power settings. This finding is consistent across included studies^[^14^].^ This is implicit, considering the lower energy delivered may result in longer time needed for tissue incision and hemostasis. Additionally, the learning curve for use of low-power settings may be more, due to the need for more precision^[^22^]^.

Low-power settings required a higher irrigation fluid volume intraoperatively as well. This in consistent with the longer operative times, thus needing higher irrigation. This is consistent across included studies as well^[^5^]^. Despite the higher irrigation and operative times, extracted tissue volume remained same across both groups, suggesting that efficiency of the procedure remained somewhat similar across high- and low-power laser settings.

Delayed complications such as urethral stricture and BNC also merit consideration, as these may develop several months after LEP and are influenced by factors such as energy delivery, surgical technique, and tissue handling. Recent prospective and registry-based studies have reported late stricture rates of 1–3% and BNC rates below 1% following HoLEP, with no clear difference between high- and low-power systems when performed by experienced surgeons^[^23,24^]^. Likewise, emerging robotic approaches, including robotic simple prostatectomy and robotic-assisted enucleation, have been proposed as alternatives for large glands or in centers with extensive robotic expertise. Comparative analyses show that robotic simple prostatectomy offers similar functional outcomes but at the cost of longer hospital stay, higher perioperative morbidity, and substantially greater resource utilization compared with HoLEP^[^25^]^. Current evidence therefore suggests that while robotic surgery may provide ergonomic and visualization advantages, LEP, irrespective of power setting, remains superior in terms of efficiency, cost-effectiveness, and durability of outcomes.

Clinically, the modestly longer operative and enucleation times observed with LP-HoLEP must be weighed against the patient-centered benefits of improved QoL scores and lower post-void residuals. Longer theater time can increase anesthetic exposure, resource utilization, and turnover pressures in high-volume centers, and may amplify the impact of the procedure’s learning curve on outcomes. However, recent long-term and learning-curve analyses show that enucleation efficiency, operative time, and laser-energy efficiency improve substantially with experience such that initial time penalties diminish as proficiency develops^[^22,26^]^. Furthermore, centers reporting careful technique adaptation with lower power settings have documented comparable enucleation volumes and durable functional gains, suggesting that the trade-off of extra minutes in theater may translate to meaningful reductions in PVR and patient symptoms with minimal lasting penalty. These considerations argue for individualized decision-making: in low-volume centers or early in a surgeon’s HoLEP learning curve, higher-power settings or *en bloc* technical refinements that shorten enucleation time may be preferable to limit operative duration, whereas high-volume surgeons or those aiming to prioritize minimization of postoperative irritation and PVR may reasonably adopt low-power protocols while accepting longer procedure times that tend to shorten with experience. Future trials should therefore report surgeon case experience and learning-curve metrics alongside patient-reported outcomes to allow clinicians to tailor energy settings to both institutional constraints and patient priorities.

Limitations of this study include the limited number of included studies and patients. Additionally, heterogeneity across certain outcomes limited the generalizability of our results, although heterogeneity was resolved through sensitivity analyses. Furthermore, long-term outcomes could not be quantified due to the lack of sufficient evidence, and insufficient data on surgeon experience and skill levels may have influenced clinical results. The study did not explore reasons for patient refusal of HoLEP, which are often related to a preference for less invasive treatments or concerns about cost and insurance coverage. Only four randomized controlled trials (*n* = 394) were eligible for inclusion, which inherently limits the statistical power and the robustness of the pooled estimates. In addition, considerable variability existed across studies in terms of baseline prostate volume, surgical technique, laser settings, surgeon experience, and perioperative protocols. These differences may have influenced operative efficiency, complication rates, and functional outcomes, thereby restricting the generalizability of our conclusions. The heterogeneity in gland size is particularly important, as energy requirements and enucleation dynamics differ substantially between small and large prostates. Such methodological and clinical variability underscores the need for large, multicenter, prospective randomized trials with standardized operative protocols and uniform follow-up schedules to more definitively assess the comparative effectiveness of low- versus high-power LEP across diverse patient populations. Further randomized trials are needed, particularly those conducted across a diverse cohort of patients. Longitudinal studies may help assess the efficacy of both procedures across a longer follow-up period. Subjective evaluation of surgeons on both procedures may help understand the ease and comfortability of physicians when using each technique.

## Conclusion

This meta-analysis concluded a greater improvement in QoL on use on HP-HoLEP settings. On the other hand, low-power settings resulted in longer operative and enucleation times and greater irrigation volume needed. Further trials are necessary to quantify the efficacy of each technique across a longer follow-up period.

## Data Availability

All data relevant to the study are included in the article or uploaded as supplementary information. Additional data related to this study are available from the corresponding author upon reasonable request.

## Research registration unique identifying number (UIN)

Prospectively registered via PROSPERO^[^9^]^ with the identification number CRD420251036533.
